# Complete Genome Sequence of Lacticaseibacillus rhamnosus DM065, Isolated from the Human Oral Cavity

**DOI:** 10.1128/mra.00899-22

**Published:** 2022-11-02

**Authors:** Do-Young Park, Jiyoung Hwang, Yunji Kim, Young-Youn Kim, Hye-Sung Kim, Inseong Hwang

**Affiliations:** a DOCSmedi Co., Ltd., Goyang-si, South Korea; b Apple Tree Institute of Biomedical Science, Apple Tree Medical Foundation, Goyang-si, South Korea; University of Maryland School of Medicine

## Abstract

The 3.0-Mb complete genome of Lacticaseibacillus rhamnosus strain DM065, which was isolated from the oral cavity of healthy volunteers in South Korea, was sequenced using a combination of PacBio and Illumina technologies. The genome consists of one circular chromosome and two plasmids and lacks antimicrobial resistance genes.

## ANNOUNCEMENT

To establish an oral probiotic strain library, we obtained human tongue coating samples from healthy South Korean donors between September 2021 and December 2021, which were spread on a de Man, Rogosa, and Sharpe (MRS) agar plate for 24 h at 37°C (1). A colony inhibiting oral pathogens, such as Porphyromonas gingivalis, Fusobacterium nucleatum, Tannerella forsythia, and Prevotella intermedia, was further selected by coculture on a MRS agar plate (2, 3), and the species was identified by 16S rRNA gene sequencing (Macrogen, Inc., South Korea) in February 2022.

For complete-genome sequencing, the isolate was anaerobically and statically cultivated for 24 h at 37°C in MRS broth. Genomic DNA (gDNA) was extracted using the Maxwell RSC system (Promega, USA) and sheared to 7 to 12 kb using the Megaruptor 3 (Diagenode, USA), followed by cleanup using AMPure PB beads (Pacific Biosciences [PacBio], USA). The Sequel system equipped with a single-molecule real-time (SMRT) cell 1M v3 tray (PacBio) was used to sequence libraries constructed using the SMRTbell Express template preparation kit v2.0 (PacBio). The Microbial Assembly protocol in SMRT Link v10.1.0.119588 (PacBio) (4) yielded an *oriC*-rotated circular chromosome and two plasmids (Table 1) from 51,476 subreads (*N*_50_, 11,063 bp; average length, 8,716 bp). To correct errors in the PacBio-assembled contigs, Illumina sequencing was also performed. The gDNA (100 ng) was sheared using Adaptive Focused Acoustics (AFA) technology (Covaris, USA), followed by end repair and size selection. Sequencing on the HiSeq X Ten system (Illumina, USA) was conducted with paired-end library construction using a TruSeq Nano DNA high-throughput library preparation kit v2.0 (Illumina), which yielded a total of 12,257,808 reads with Phred scores of >30 in ≥90% of each read (5, 6). Low-quality and short reads, adapter sequences, and terminal bases were filtered using Trimmomatic v0.38 (7), followed by alignment against the three PacBio circular contigs using BWA-MEM v0.7.17 (8). The final Illumina-based polishing was performed using three rounds with Pilon v1.21 (9) with a minDepth parameter of 0.01.

**TABLE 1:** Summary of assembly and annotation statistics for L. rhamnosus DM065

Genetic element	Length (bp)	GC content (%)	No. of coding sequences	No. of rRNAs	No. of tRNAs	Sequencing depth (×)	GenBank accession no.
Chromosome	2,945,770	46.7	2,658	15	60	128.3	CP102716.1
Plasmid pLRDM065A	46,212	43.6	48			136.2	CP102717.1
Plasmid pLRDM065B	28,719	40.3	35			264.9	CP102718.1
Total	3,020,701	46.6	2,741	15	60	129.7	

Genome annotation using PGAP v6.2 (10) reported 2,741 protein-coding genes, 15 rRNA genes, and 60 tRNA genes. OrthoANI (11) analysis indicated Lacticaseibacillus rhamnosus ATCC 8530 (GenBank accession number CP003094.1) as the closest strain, with 99.87% sequence identity. ResFinder v4.1 (12) and CARD v3.2.3 (13) revealed that DM065 lacks antimicrobial resistance genes. All tools were run with default parameters unless otherwise specified.

The human oral samples used in this study were provided by the Biobank of Apple Tree Dental Hospital after approval from the public institutional review board (http://public.irb.or.kr) (approval number P01-202111-31-002).

### Data availability.

The 16S rRNA gene sequence, the genome sequence, and the raw sequencing reads for DM065 were deposited in GenBank under accession numbers OP579173.1, CP102716.1, CP102717.1, and CP102718.1, BioProject accession number PRJNA851917, BioSample accession number SAMN29255128, and SRA accession numbers SRX15898508 and SRX15898509.
